# Five-year mortality in a cohort of people with schizophrenia in Ethiopia

**DOI:** 10.1186/1471-244X-11-165

**Published:** 2011-10-10

**Authors:** Solomon Teferra, Teshome Shibre, Abebaw Fekadu, Girmay Medhin, Asfaw Wakwoya, Atalay Alem, Gunnar Kullgren, Lars Jacobsson

**Affiliations:** 1 Department of Psychiatry, School of Medicine, College of Health Sciences, Addis Ababa University, Addis Ababa, Ethiopia; 2 Aklilu Lemma Institute of Pathobiology, College of Health Sciences, Addis Ababa University, Addis Ababa, Ethiopia; 3 Amanauel Specialized Mental Hospital, Addis Ababa, Ethiopia; 4 Division of Psychiatry, Department of Clinical Sciences, Umeå University, Umeå, Sweden; 5 Department of Psychological Medicine, Section of Neurobiology of Mood Disorders, Institute of Psychiatry, King's College London, UK

## Abstract

**Background:**

Schizophrenia is associated with a two to three fold excess mortality. Both natural and unnatural causes were reported. However, there is dearth of evidence from low and middle income (LAMIC) countries, particularly in Africa. To our knowledge this is the first community based report from Africa.

**Methods:**

We followed a cohort of 307 (82.1% males) patients with schizophrenia for five years in Butajira, rural Ethiopia. Mortality was recorded using broad rating schedule as well as verbal autopsy. Standardized Mortality Ratio (SMR) was calculated using the mortality in the demographic and surveillance site as a reference.

**Result:**

Thirty eight (12.4%) patients, 34 men (11.1%) and 4 women (1.3%), died during the five-year follow up period. The mean age (SD) of the deceased for both sexes was 35 (7.35). The difference was not statistically significant (p = 0.69). It was 35.3 (7.4) for men and 32.3 (6.8) for women. The most common cause of death was infection, 18/38 (47.4%) followed by severe malnutrition, 5/38 (13.2%) and suicide 4/38 (10.5%). The overall SMR was 5.98 (95% CI = 4.09 to7.87). Rural residents had lower mortality with adjusted hazard ratio (HR) of 0.30 (95% CI = 0.12-0.69) but insidious onset and antipsychotic treatment for less than 50% of the follow up period were associated with higher mortality, adjusted HR 2.37 (95% CI = 1.04-5. 41) and 2.66(1.054-6.72) respectively.

**Conclusion:**

The alarmingly high mortality observed in this patient population is of major concern. Most patients died from potentially treatable conditions. Improving medical and psychiatric care as well as provision of basic needs is recommended.

## Background

Schizophrenia is often called a 'life shortening disease' [1]. Mortality is increasingly recognized as a crucial outcome measure for quality of care patients with schizophrenia receive [2,3]. Several studies reported that people with schizophrenia live shorter lives compared with the general population [4]. Most of the reports come from inpatients registers, death registers or cohort of patients. These have found a two to three fold increase in all cause mortality compared with the general population, moreover; when compared with other mental disorders, having the diagnosis of schizophrenia carried a higher risk of mortality [5-9]. Factors contributing to premature death in people with schizophrenia include suicide, untreated somatic conditions, and adverse effects of psychotropic medications to mention a few [10]. A recent study which compared survival status of people with diabetes in the UK showed that having a diagnosis of schizophrenia increased the risk of death from diabetes by almost two fold [11].

Most of the reports come from centers in industrialized countries. Although there is paucity of data from low and middle income (LAMIC) countries, the existing data shows that there is high level of mortality in patients with schizophrenia from these countries with pooled Standardized Mortality Ratios (SMR) comparable with those patients who live in industrialized countries [12]. The notion that schizophrenia has a benign course and outcome in LAMIC countries has been challenged by several reports which showed poor outcome for patients from these countries. These reports emerge largely from countries in Africa, Asia and Latin America which are called LAMIC countries [13,14]. Reports of better outcome in LAMIC countries have not taken mortality into account.

The aim of this study was to determine the five- year mortality in a rural community based cohort with schizophrenia and to describe the causes of death as well as to suggest some interventions for potentially modifiable risk factors. We hypothesized that the cohort in our study would have high level of mortality. In addition to the general report of excess mortality, our hypothesis was based on reports that associate poor clinical outcome with increased risk of mortality [15]. Our cohort was previously reported to have poor short-term clinical outcome with a large proportion of patients having had chronic or relapsing course [16].

## Methods

### Study site and Establishment of the cohort

Details of the study settings and methods screening and diagnostic methods and establishment of the cohort can be found in a previously published paper [16]. The study was done in Butajira district, which houses a demographic surveillance site (DSS), located 132 km south of the capital city, Addis Ababa. The presence of the DSS offered a unique opportunity to calculate age and sex standardized mortality ratios.

The cohort was identified through a two-stage sampling design: an initial screen to identify potential cases with severe mental disorders (schizophrenia, bipolar disorder and depressive disorder) and a second confirmatory assessment stage. The initial screen targeted the entire adult population of the 44 sub-districts, ages 15-49, estimated to be 83,282 [17]. This initial screen involved a supervised, door-to-door survey of the target population using the affective and psychoses modules of the Composite International Diagnostic Interview (CIDI), version 2.1 [18]. The CIDI was administered to 68,378 individuals (82% of the target population). Key informants were also employed to augment the screening with the CIDI interview. In the second stage, confirmatory diagnostic assessment was conducted for all CIDI positives using SCAN version 2.1 [19].

The following three criteria were selected for inclusion: age between 15 and 49 years; residence of at least 6 months in the study area prior to inclusion and meeting criteria for the diagnosis of schizophrenia according to ICD-10 [20] after a SCAN interview.

A total of 2,878 individuals were identified as positive for the psychosis and mood section through the initial screen and 2,285 (79.4%) volunteered for the SCAN interview. Of those interviewed with SCAN, 321 were diagnosed as having schizophrenia.

### Follow-up and confirmation of mortality

Field workers employed by the research project were assigned to each patient to facilitate the follow up. Patient's monthly clinical status was recorded by either a psychiatry resident or an experienced psychiatry nurse. Annual SCAN was done by the psychiatry resident. The cohort was followed-up for the duration of 5 years. Deaths were identified by the field workers and the causes for death were established by physicians through direct interviews of close relatives and local health workers when appropriate. The clinical and functional status of the deceased patients around the time of death was recorded by a psychiatry resident. Both broad rating schedule and verbal autopsy questionnaires were used to gather information as required by the situation [21,22]. The broad rating schedule was developed by the WHO for recording broad range of information including severity of symptoms and functioning from multiple sources including informants and records and can be used for cases lost to follow-up as well as deceased cases [22].

### Data management and analysis

Full time data entry clerk first checked all interview forms in the field for completeness, accuracy and consistency. Epi-info program (version 6.2) was used for data entry. Double data-entry and consistency checks were employed to assure the quality of data entry.

Stata version 11 (Stata Corp) was used for both descriptive analysis and survival analysis. The age and sex standardized mortality ratio was calculated using mortality in the DSS as a reference [23]. Cox regression was used to evaluate the effect of socio-demographic and clinical characteristics on time to death.

### Ethical Considerations

Ethical approval of the study was obtained from the Faculty of Medicine, Addis Ababa University. All participants provided consent to be interviewed. For the cases identified by the research a psychiatric clinic was established which provided free medication during the follow-up period.

## Result

### Demographic characteristics of the deceased

The initial cohort comprised a total of 321 cases with schizophrenia. We had complete data for 307 of the 321 patients (95.6%) that were included in the initial cohort, 252 (82.1%) males and 55 (17.9%) females at the time of analysis of the 5-year follow up. The majority were married (51.8%), had no formal education (53.7%), Muslim by religion (74.6%) and were from a rural setting (76.8%) (Table 1).

**Table 1:** Sociodemographic characteristics of the cases with schizophrenia in Butajira, Ethiopia

Characteristics	Male		Female		Total	
	
	(deceased) n = 252 (34)	% (deceased) 82.1 (13.5)	(deceased) n = 55 (4)	%(deceased) 17.9(7.3)	(deceased) n = 307(38)	% (deceased) 100(12.4)
Age (307)*						
15-24	39 (4)	15.5 (1.6)	18 (1)	32.7(1.8)	57(5)	18.6 (1.6)
25-34	106 (14)	42.1 (5.6)	17(2)	30.9(3.6)	123(16)	40.1(5.2)
35-49	73(16)	29 (6.3)	16(1)	29(1.8)	89(17)	29 (5.5)

Marital Status (307)						
Single	67(8)	26.6 (3.2)	11(2)	20 (3.6)	78 (10)	25.4(3.3)
Married	116(21)	46.0 (8.3)	21(1)	38.2(1.8)	137(22)	44.6(7.2)
Other	35(5)	13.9(2)	19(1)	34.5(1.8)	54(6)	17.6(2)

Education(307)						
No formal education	103(21)	40.9(8.3)	38(3)	69.1(5.5)	141(24)	45.9(7.8)
Elementary	73(8)	29(3.2)	8(1)	14.5(1.8)	81(9)	26.4(2.9)
Education	42(5)	16.7(2)	5(0)	9.1(0)	47(5)	15.3(1.6)
Secondary and above						

Religion (307)						
Muslim	172(25)	68.3(9.9)	30(2)	54.5(3.6)	202(27)	65.8(8.8)
Christian	46(9)	18.3(3.6)	21(2)	38.2(3.6)	67(11)	21.8(3.6)

Residence (307)						
Urban/Semiurban	41(12)	16.3(4.8)	16(2)	29.1(3.6)	57(14)	18.6(4.6)
Rural	177(22)	70.2(8.7)	35(2)	63.6(3.6)	212(24)	69(7.8)

*data was available for 307 cases though cases identified in the original cohort were 321.

### All cause mortality

A total of 38 (12.4%) patients, thirty four men (11.1%) and four women (1.3%), died during the 5-year follow-up period. The mean age (SD) of the deceased for both sexes was 35 (7.4). It was 35.3 (7.4) for men and 32.3 (6.8) for women. The difference was not statistically significant (p = 0.69).

### Cause and place of death

The most common cause of death was infection such as malaria, tuberculosis and severe diarrheal disease 18/38 (47.4%) followed by severe malnutrition 5/38 (13.2%) and suicide 4/38 (10.5%). The suicide cases were exclusively men. One of them committed suicide by setting fire on himself and burnt to death. One male and one female patient were hit by running cars and died. One of them died immediately on the road while the other was taken to a clinic but couldn't be saved. The causes of death for three (7.9%) were undetermined. One of these was a homeless person who was known to have been drinking and smoking excessively. He was found dead in an abandoned house and was presumed to have died from natural causes, presumably from the complication of his habit of heavy alcohol drinking. The other two reportedly died suddenly, and we were unable to establish causes of death for these.

Place of death for the majority (78.9%) was at home. For more than half of the deceased their death was either caused or related with their mental illness. Only 2 of the cases were in remission during the time of death and only 3 had a GAF score of 60 or more (Table 2).

**Table 2:** Status of the schizophrenia cohort after 5 years of follow-up in Butajira, Ethiopia

Characteristics	Men (N = 252)		Women (N = 55)		Total (N = 307)	
	
	No.	%	No.	%	No.	%
Alive: completed 5-year follow up	180	71.4	42	13.7	222	72.3

Refusal	9	3.6	0	0	9	2.9

Out migrated	9	3.6	2	0.65	11	3.6

Vagrant	6	2.4	1	0.32	7	2.3

Untraceable	1	0.4	0	0	1	0.3

Deceased:	34	13.5	4	7.3	38	12.4

Infection: malaria, TB...	15	44.1	3	75	18	47.4

Severe malnutrition	5	14.7	0	0	5	13.2

Suicide	4	11.8	0	0	4	10.5

Hit by Car	1	2.9	1	25	2	5.3

Other natural causes	6	15.8	0		6	15.9

Unknown	3	7.9			3	7.9

Relation with mental illness						

Caused	9	26.5	0	0	9	23.7

Related	11	32.4	1	25	12	31.6

Independent	9	26.5	2	50	11	28.9

Undetermined	5	14.7	1	25	6	15.8

Place of death						

Home	27	79.4	3		30	78.9

Health facility	2	5.9	1		3	7.9

Other places	5	14.7	0		5	13.2

GAF score around death						

< 60	27	79.4	2	50	29	76.3

≥ 60	2	5.9	1	25	3	7.9

unknown	5	14.7	1	25	6	15.8

MSE around death						

Remission	2	5.9	0	0	2	5.3

Partial remission	7	20.6	1	25	8	21.0

In episode	23	67.6	3	75	26	68.4

Undetermined	2	5.9	0	0	2	5.3

### Mortality rate and standardized mortality ratio (SMR)

The mortality rate for study population was 8,661/100,000 for men (mortality rate for the reference population = 2,202.8/100,000) and 5,006/100,000 for women (reference population = 1,802/100,000). The overall age and sex standardized mortality ratio (SMR) was 5.98 (95% CI = 4.09-7.87). The total SMR was 6.27(95% CI = 4.16-8.38) for men and 4.3 (95% CI = 1.02-8.52) for women (Table 3).

**Table 3:** Age and Sex Standardized Mortality Ratio (SMR) of patients with schizophrenia in Butajira, Ethiopia*

Age Group	Men				Women			
	
	Observed Deaths	Expected death	Observed/Expected Mortality (SMR)	95% CI	Observed Deaths	Expected death	Observed/Expected Mortality (SMR)	95% CI
15-24	4	0.73	5.48	0.11-10.85	1	0.19	5.26	5.05-15.57

25-34	14	2.23	6.28	2.99-9.57	2	0.29	6.9	-2.66-16.5

35-49	16	2.95	5.42	2.76-8.18	1	0.53	1.89	-1.81-5.59

Total	34	5.42	6.27	4.16-8.38	4	0.93	4.3	1.02-8.52

*Overall SMR = 5.98 (95% CI = 4.09-7.87)

### Correlates of Mortality

**Figure 1:**
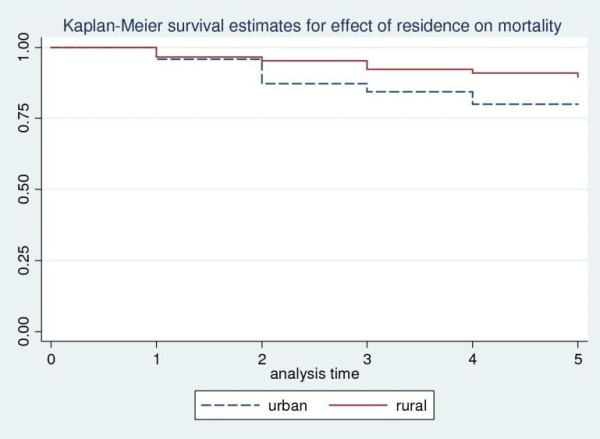
**Kaplan-Meier Curve showing survival probability and place of residence in people with schizophrenia: a five-year follow-up in Butajira, Ethiopia**.

**Figure 2:**
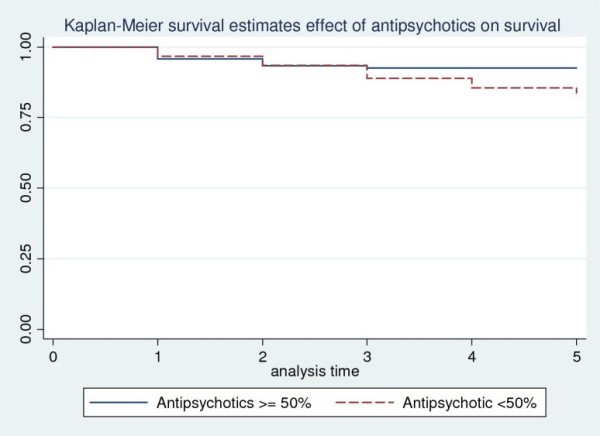
**Kaplan-Meier Curve showing survival probability and treatment with antipsychotics in people with schizophrenia: a five-year follow-up in Butajira, Ethiopia**.

In a Cox regression analysis of time to death where socio-demographic and clinical correlates were include as predictor variables urban/semi-urban residence (Figure-1) and insidious onset of illness were significantly associated with increased risk of mortality with adjusted hazard ratio (HR) for rural residence of 0.30 (95% CI = 0.12-0.69) and adjusted HR for insidious onset of 2.37 (95% CI = 1.04-5. 41). Treatment with antipsychotics for less than 50% of the follow-up time (Figure-2) was also significantly associated with higher mortality with adjusted HR of 2.66(95%CI: 1.054-6.72) (Table 4). The increased mortality among urban dwellers and those patients who were not taking antipsychotic medications for at least 50% of the follow-up period is illustrated by the Kaplan-Meier survival curves in Figures 1 and 2.

**Table 4:** Association of mortality in schizophrenia with sociodemographic and clinical factors using Cox regression model, Butajira Ethiopia

Characteristics	Crude HR (95%CI)	P-value	Adjusted HR (95% CI)	P-value
Sex				

Male	ref			

Female	0.54 (0.19-1.52)	0.244		

Age Group				

15-24	ref		ref	

25-34	1.49 (0.55-4.09)	0. 43	1.85(0.46-7.43)	0.38

35-50	2.03 (0.75-5.51)	0.16	3.99(0.73-21.69)	0.11

Marital status				

Single	ref		ref	

Married	1.21 (0.57-2.56)	0.61	3.04(0.98-9.40)	0.053

Other	0.86 (0.31-2.37)	0.77	1.36(0.38-4.83)	0.633

Educational Status				

No formal	ref		ref	

Elementary	0.67 (0.31-1.44)	0.31	0.76(0.30-1.93)	0.56

Secondary	0.64 (0.24-1.68)	0.37	0.36(0.11-1.22)	0.10

Place of residence				

Urban	ref		ref	

Rural	0.49 (0.26-0.96)	0.038	0.34(0.14-0.80)	0.014*

Age of onset				

≤ 19	ref		ref	

20-29	1.64 (0.69-3.88)	0.26	1.15(0.36-3.71)	0.81

≥ 30	1.78 (0.65-4.92)	0.26	1.07(0.24-4.67)	0.93

Duration of illness				

≤ 2 yrs	ref		ref	

> 2 yrs	1.38 (0.61-3.16)	0.44	0.73(0.25-2.13)	0.56

Speed of onset				

Acute	ref		ref	

Insidious	2.22 (1.04-4.72)	0.038	2.86(1.24-6.57)	0.013*

Schizophrenia subtype				

Paranoid	ref		ref	

Undifferentiated	1.09 (0.49-2.45)	0.82	0.66(0.25-1.75)	0.40

Other	1.52 (0.69-3.34)	0.30	0.90(0.33-2.43)	0.84

Antipsychotics 50%				

≥ 50%	ref		ref	

< 50%	2.12 (1.02-4.55)	0.044	2.66(1.054-6.72)	0.038*

* P < 0.05 significant difference

## Discussion

Our report on mortality in schizophrenia is one of the few population based studies coming from LAMIC countries. Even most reports from high-income countries on schizophrenia mortality come from hospital based studies. Participants of our cohort were predominantly treatment naïve at the time of enrolment identified through a house-to-house population survey. A very rigorous follow-up of patients on monthly basis enabled us to determine the mental status of our study participants and their functioning around the time of death. It was also possible to describe the possible causes of death using verbal autopsy [21]. The presence of a DSS site enabled us to calculate the SMR [23].

### Mortality

It is a well established fact that people with schizophrenia live shorter than their healthy counterparts, having a 2-3 fold increase in mortality [1,4,12]. In our study which reported a five-year mortality, we found a six fold increase in overall mortality (overall SMR = 5.98); men had the highest mortality (SMR = 6.27) compared with females (SMR = 4.3), and it was much higher than what has been reported from different countries so far. The absolute number of death was also one of the highest in the world, 12% of the patients died in 5 years. The mean age of death was also very low: men died approximately 14 years and women 20 years younger than the reference population. Life expectancy at birth was 49.3 and 52. 3 years for men and women respectively [23].

It is worth mentioning here that direct comparison with other reports may not be appropriate given the difference in sampling methods and the study setting such as use of hospitalized patients. Caution is needed when comparing SMRs that come from different populations as it might be affected by the mortality rate in the reference population [24]. But, there are some community based reports which we can make a reasonable comparison with. A community based 10 year mortality from China reported an overall SMR of 4.0 (95% CI = 2.4-5.8). Similar to our cohort males had higher SMR than females [25]. One main difference in the current study and the China study is the duration of follow-up. In the current study we are reporting the 5- year follow up and the length of the follow-up time may contribute to higher mortality as most patients die during early stages of follow up [26,27]. Another study from a LAMIC country (Bali) which reported a similar SMR to ours (5.98) was based on hospitalized patients; similar to our finding, men had higher SMR (7.11) than women (4.05) [28]. However, other reports from LAMIC countries reported low rates of mortality. For instance, a study from Taiwan, which came from a linkage data of a large sample of inpatients, reported SMR of 2.70 [29]. In the WHO multi-center study that reported a 15- and 25-year follow-up of cases from Chandigarh (Rural and urban), Agra and Cali, the SMR was around 2-3, but a high proportion of drop-out rate, as high as 40 percent in some sites, could be one reason for the low mortality in these studies [24].

### Probable causes of death

Not surprisingly, infectious causes were the most common causes of death in our schizophrenia cohort unlike reports from high-income countries for which non-communicable diseases such as cardiovascular diseases, neoplasms, metabolic disturbances and possibly psychotropic medications and smoking related mortality [6,8,27,30]. Butajira has diverse climate and topography ranging from low land to high land. There are no special disease conditions peculiar to the district. As it is typical for the country, infectious diseases are common in the district including malaria [31], TB and HIV [32] contributing to 53% of the overall mortality that occurred in the Butajira DSS site [33]. Interestingly, despite the HIV pandemic, there is a trend of decline in mortality observed in young people in the Butajira DSS in the past 18 years [34]. We had no data regarding the HIV status of our cohort.

The district is prone to periodic draught leading to food shortage, but the severe malnutrition observed in the patient groups is more than what one would see in the general adult population suggesting possible neglect (or preferential treatment of healthy groups of the family in times of shortage).

Well designed studies describing causes of mortality in schizophrenia are lacking in Africa. Two hospital based 10 year mortality reports from Nigeria found that the commonest cause of death was infectious as was found in our study [35,36]. It is not possible to estimate general mortality related to schizophrenia based on the two Nigerian studies because they didn't report specific diagnosis.

Unfortunately, we couldn't calculate the cause specific SMR because of lack of reference data in the area. This limits comparison of our finding with other studies. However, the crude figure among patients with schizophrenia dying from unnatural causes, i.e. suicide and accident (15.8%), was much higher than the proportion who died from injuries over a five year period of observation in a subsample of the general population within the Butajira district (1.7%) [21]. It is well known that people with schizophrenia have high rates of suicide compared with the general population [37,38]. The proportion of patients who committed suicide was higher than reports from other LAMIC countries [39]. Interestingly, all the suicide cases were men unlike reports from mainland China where more females with schizophrenia commit suicide [40].

### Mortality as an outcome measure

Mortality has been mentioned as an important measure of outcome in the course of schizophrenia [3,41]. There has been a widespread belief that schizophrenia runs a benign course in LAMIC countries. This belief stems from the WHO multicenter studies which reported a favorable outcome for patients living in developing countries [22,24,42,43]. This belief prevailed for a long time until reports started to emerge from LAMIC countries which challenged the idea that schizophrenia might not run a benign course in these countries [13,14,25]. Short term outcome from our cohort showed that most of our patients run a chronic non-remitting course [16]. A predominantly chronic non-remitting course coupled with high mortality suggests a poor course and outcome in this setting.

In this study we found that those who had insidious onset had higher rate of mortality, whereas, those who lived in rural areas had less mortality compared with those living in urban areas. Treatment with antipsychotic medication for at least 50% of the follow-up time was associated with lower risk of mortality. In our earlier report on course and outcome, treatment was the most important predictor of good clinical course and outcome. So, ensuring continued treatment with antipsychotics was found to improve clinical course and outcome as well as reduce mortality in this cohort [16].

### Strength and limitation of the study

To our knowledge this is the first community based report on mortality in schizophrenia from the African continent which adds to the knowledge base regarding the mortality situation in schizophrenia worldwide. The study was based on a mixed prevalence cohort consisting of both acute and chronic cases and the majority of the cohort had illness longer than two years. Having a large number of chronic cases at enrolment was considered a limitation as these patients would die early in the course of the follow up period, but 'nature of onset' rather than 'duration of illness' was associated with increased mortality in our cohort. The other limitation was the information obtained on the cause of death, which mostly relied on information provided by family members. Post mortem examination data was not available. Furthermore it was not possible to check the medical records of patients as most of them died at home. Yet another limitation is the lack of physical health checkup for patients in our follow-up protocol which was only restricted to the time of enrolment.

## Conclusion

A six fold increase in mortality was noted compared with the reference population in the area, an alarmingly high level of mortality. Men were particularly vulnerable to mortality as evidenced by the highest number of death from both natural and unnatural causes. Furthermore most patients died from potentially treatable conditions such as infections due to malaria and TB and malnutrition. Unnatural causes such as suicide and accident were also important causes of death in this patient population. Suicide was a very important cause of death in the male patients. Another striking finding is that the majority of patients died at home which suggests a possible neglect of these patients. The presence of malnutrition as a cause death in these patients suggests a possible discrimination and neglect at family level. Again, the majority of patients were in severe psychotic episode which showed the importance of the disorder they had on causing their death. Patients need to have regular medical evaluation and treatment besides psychiatric care as most patients died from treatable medical conditions. They also need to have their basic needs met such as availability food to prevent malnutrition. Family and community education needs to be intensified. Ensuring continued treatment with antipsychotics is also a feasible intervention to reduce mortality in this setting.

## Competing interests

The authors declare that they have no competing interests.

## Authors' contributions

ST, TS and AW carried out the data collection, participated in the analysis and drafted the manuscript. TS, AF, AA, GK, and LS participated in the design of the study and ST and GM performed the statistical analysis. TS, AF, AA, GK and LS conceived of the study, and participated in its design and coordination. All authors read and approved the final manuscript.

## Pre-publication history

The pre-publication history for this paper can be accessed here:

http://www.biomedcentral.com/1471-244X/11/165/prepub

## References

[B1] Allebeck P Schizophrenia: A Life-Shortening Disease Schizophr Bull 1989 15 81 89 2717890 10.1093/schbul/15.1.81

[B2] Drew LR Mortality and mental illness Aust N Z J Psychiat 2005 39 194 197 10.1080/j.1440-1614.2005.01543.x 15701070

[B3] Seeman MV An Outcome Measure in Schizophrenia: Mortality Can J Psychiatry 2007 52 55 60 17444079 10.1177/070674370705200109

[B4] Brown S Excess mortality of schizophrenia: A meta-analysis Br J Psychiatry 1997 171 502 508 10.1192/bjp.171.6.502 9519087

[B5] Mortensen PB Juel K Mortality and causes of death in first admitted schizophrenic patients Br J Psychiatry 1993 163 183 189 10.1192/bjp.163.2.183 8075909

[B6] Brown S Barraclough B Inskip H Causes of the excess mortality of schizophrenia Br J Psychiatry 2000 177 212 7 10.1192/bjp.177.3.212 11040880

[B7] Osby U Correia N Brandt L Ekbom A Spare'n P Mortality and causes of death in schizophrenia in Stockholm county, Sweden Schizophr Res 2000 45 21 28 10.1016/S0920-9964(99)00191-7 10978869

[B8] Joukamaa M Heliövaara M Knekt P Aromaa A Raitasalo R Mental disorders and cause-specific mortality Br J Psychiatry 2001 179 498 502 10.1192/bjp.179.6.498 11731351

[B9] Heila H Haukka J Suvisaari J Lönnquist J Mortality among patients with schizophrenia and reduced hospital care Psychol Med 2005 35 725 32 10.1017/S0033291704004118 15918349

[B10] Auquier P Lancon C Rouillon F Lader M Holmes C Mortality in schizophrenia Pharmacoepidemiol Drug Saf 2006 15 873 879 10.1002/pds.1325 17058327

[B11] Vinogradova Y Coupland C Hippisley-Cox J Whyte S Penny C Effects of severe mental illness on survival of people with diabetes Br J Psychiatry 2010 197 272 277 10.1192/bjp.bp.109.074674 20884949

[B12] Saha S Chant D McGrath J A systematic review of mortality in schizophrenia: is the differential mortality gap worsening over time? Arch Gen Psychiatry 2007 64 1123 1131 10.1001/archpsyc.64.10.1123 17909124

[B13] Patel V Cohen A Thara R Gureje O Is the outcome of schizophrenia really better in developing countries? Rev Bras de Psiquiatr 2006 28 149 152 10.1590/S1516-44462006000200014 16810400

[B14] Cohen A Patel V Thara R Gureje O Questioning an Axiom: Better Prognosis for Schizophrenia in the Developing World? Schizophr Bull 2008 34 229 244 17905787 10.1093/schbul/sbm105 PMC2632419

[B15] Mojtabai Varma VK Malhotra V Mattoo SK Misra AK Mortality and long-term course in schizophrenia with a poor 2-year course: a study in a developing country Br J Psychiatry 2001 178 71 75 10.1192/bjp.178.1.71 11136214

[B16] Alem A Kebede D Fekadu A Shibre T Fekadu D Clinical course and outcome of schizophrenia in a predominantly treatment-naïve cohort in rural Ethiopia Schizophr Bull 2009 35 646 654 10.1093/schbul/sbn029 18448478 PMC2669573

[B17] OPHCC The 1994 Population and Housing Census of Ethiopia. Results for Southern Nations, Nationalities and Peoples' Region. Abridged Statistical Report 1996

[B18] WHO Composite International Diagnostic Interview, Core Version, 2.1 1997 Geneva; World Health Organization

[B19] WHO Schedules for Clinical Assessment in Neuropsychiatry, Version 2.1 1997 Geneva; World Health Organization

[B20] WHO The ICD-10 Classification of Mental and Behavioural Disorders: Clinical descriptions and diagnostic guidelines 1992 Geneva; World Health Organization

[B21] Lulu K Berhane Y The use of simplified verbal autopsy in identifying causes of adult death in a predominantly rural population in Ethiopia BMC Public Health 2005 5 58 10.1186/1471-2458-5-58 15935096 PMC1164421

[B22] Sartorius N Gulbinat W Harrison G Laska E Siegel C Long-term follow-up of schizophrenia in 16 countries. A description of the International Study of Schizophrenia conducted by the World Health Organization Soc Psych Psych Epid 1996 31 249 258 10.1007/BF00787917 8909114

[B23] Berhane Y Byass P Site description: physical geography of the Butajira DSA. Butajira DSS; Ethiopia http://paulaclark.name/CV/BOOK%20CHAPTERS/population%20health%20and%20survival%20at%20indepth%20sites.html

[B24] Harrison G Hopper K Craig T Laska E Siegel C Recovery from psychotic illness: a 15- and 25-year international follow-up study Br J Psychiatry 2001 178 506 517 10.1192/bjp.178.6.506 11388966

[B25] Ran MS Chen EY Conwell Y Chan CL Xiang YM Mortality in People with Schizophrenia in Rural China: 10-Year Cohort Study Br J Psychiatry 2007 190 237 242 10.1192/bjp.bp.106.025155 17329744

[B26] Craig TJ Ye Q Bromet EJ Mortality among first-admission patients with psychosis Compr Psychiatry 2006 47 246 251 10.1016/j.comppsych.2005.11.004 16769297

[B27] Montout C Casadebaig F Lagnaoui R Verdoux H Philippe A Begaud B Neuroleptics and mortality in schizophrenia: prospective analysis of deaths in a French cohort of schizophrenic patients Schizophr Res 2002 57 147 156 10.1016/S0920-9964(01)00325-5 12223245

[B28] Kurihara T Kato M Kashima H Takebayashi T Reverger R Excess mortality of schizophrenia in the developing country of Bali Schizophr Res 2006 83 103 105 10.1016/j.schres.2006.01.018 16531012

[B29] Chen WJ Huang Y-J Yeh L-L Rin H Hwu H-G Excess mortality of psychiatric inpatients in Taiwan Psychiat Res 1996 62 239 250 10.1016/0165-1781(96)02875-2 8804134

[B30] Brown S Kim M Mitchell C Inskip H Twenty-five year mortality of a community cohort with schizophrenia Br J Psychiatrty 2010 196 116 121 10.1192/bjp.bp.109.067512 PMC4560167 20118455

[B31] Tesfaye S Belyhun Y Teklu T Mengesha T Petros B Malaria prevalence pattern observed in the highland fringe of Butajira, Southern Ethiopia: a longitudinal study Malar J 2011 10 153 10.1186/1475-2875-10-153 21649923 PMC3141588

[B32] Yassin M Takele L Gebresenbet S Lera M HIV and Tuberculosis Coinfection in the Southern Region of Ethiopia: A Prospective Epidemiological Study Scand J Infect Dis 2004 36 670 673 10.1080/00365540410020848 15370654

[B33] Fantahun M Berhane Y Ulf Högberg U Stig Wall S Peter Byass P Young adult and middle age mortality in Butajira demographic surveillance site, Ethiopia: lifestyle, gender and household economy BMC Public Health 2008 8 268 10.1186/1471-2458-8-268 18671854 PMC2519081

[B34] Molla M Byass P Berhane Y Lindtjorn B Mortality Decreases among Young Adults in Southern Central Ethiopia Ethiop J Health Dev 2008 22 218 225

[B35] Abiodun OA Mortality in a psychiatric population: a Nigerian psychiatric hospital experience Acta Psychiatr Scand 1988 77 654 657 10.1111/j.1600-0447.1988.tb05184.x 3261482

[B36] Malomo IO Aina OF Ladapo HT Owoeye AO Ten-year mortality review in a pioneer psychiatric hospital in West Africa East Afr Med J 2003 80 379 383 16167755 10.4314/eamj.v80i7.8723

[B37] Caldwell CB Gottesman II Schizophrenics Kill Themselves Too: A Review of Risk Factors for Suicide Schizophr Bull 1990 16 571 589 2077636 10.1093/schbul/16.4.571

[B38] Harris EC Barraclough B Suicide as an outcome for mental disorders. A meta-analysis Br J Psychiatry 1997 170 205 228 10.1192/bjp.170.3.205 9229027

[B39] Thara R Honriotta M Joseph A Ten-year course of schizophrenia: the Madras longitudinal study Acta Psychiatr Scand 1994 90 329 336 10.1111/j.1600-0447.1994.tb01602.x 7872036

[B40] Phillips MR Yang G Li S Li Y Suicide and the unique prevalence pattern of schizophrenia in mainland China: a retrospective observational study Lancet 2004 364 1062 68 10.1016/S0140-6736(04)17061-X 15380965

[B41] Bushe CJ Taylor M Haukka J Review: Mortality in schizophrenia: a measurable clinical endpoint J Psychopharmacol 2010 24 17 25 10.1177/1359786810382468 20923917 PMC2951589

[B42] Sartorius N Jablensky A Shapiro L Two-year follow-up of the patients included in the WHO International Pilot Study of Schizophrenia Psychol Med 1977 7 529 541 10.1017/S0033291700004517 905470

[B43] Jablensky A Sartorius N Ernberg G Anker M Korten A Schizophrenia: manifestations, incidence and course in different cultures: a World Health Organization ten-country study Psychol Med Monogr Suppl 1992 20 1 97 1565705 10.1017/s0264180100000904

